# Nanoparticle Risks and Identification in a World Where Small Things Do Not Survive

**DOI:** 10.1007/s11569-017-0305-6

**Published:** 2017-08-26

**Authors:** Erik Reimhult

**Affiliations:** Department of Nanobiotechnology, Institute of Biologically Inspired Materials, University of Natural Resources and Life Sciences, Vienna, 1190 Vienna, Austria

**Keywords:** Nanosafety, Nanorisk, Colloidal science, Nanoparticle aggregation, Particle characterization, Nanomaterials, Regulation

## Abstract

The risks of materials containing nanoscale components are in the public debate discussed as if a manufactured nanomaterial will remain invariant with time and environmental exposure, and as if we can identify its risks by the risks of its nanoscale components. Additionally, the debate on mitigation of specific nanorisks by new legislation implicitly assumes that we can have full and accurate knowledge of the distribution and composition of nanomaterials in a product or the environment. In this discussion note, I argue that physical laws intrinsic to the behavior of nanoparticles both lead to limits on the risks to which we are likely exposed and on our technological ability to verify compliance with new regulations. My conclusion is that governmental actors should be careful not to overreact in their response to a technological revolution that only in few areas is likely to lead to increased public exposure, and in ﻿doing so using legal measures for which compliance cannot be monitored.

## Nanoparticle Risks and Identification in a World Where Small Things Do Not Survive

### The Case for Considering Nanomaterial Risks

For many years, since the introduction in fiction literature of self-replicating nanobots taking over the Earth, there has been an expanding and increasingly serious discussion about the risks of widespread use of nanotechnology. After a brief spotlight in the public awareness at the turn of the millennium, the debate has since then mostly taken place within the “professional elites” of society, among scientists, health professionals, non-governmental organizations (NGOs), and political representatives. It has also focused more, and rightly so, on the risks posed by large-scale production of nanomaterials than on self-replicating nanobots.

Nanomaterials on their own or as additives to traditional polymer composite materials have started a revolution in materials science that improves performance and resource efficiency far beyond what was previously dreamed of outside science fiction books. Thus, manufacturing of various nanomaterials and their spread into our everyday lives and the environment have now started in earnest. Production costs of high-performance composite materials and nanoparticles continue to rapidly decrease through technological progress. Production and exposure, as well as waste treatment of nanomaterials, will therefore rapidly increase. This intuitive scenario should and does trigger a real debate about the risks of diverse and widespread use of nanomaterials. However, should exploding production and use automatically trigger dedicated hazard and risk debates? And, should it trigger regulations with prefixes such as: *nano*risk, *nano*toxicology, and specific restrictions on *nano*materials? The current debate is underpinned by an implicit assumption that use of nanomaterials equals public exposure to nanoparticles and nanospecific consequences; the dangers of which can and should be regulated separately. In this discussion note, I discuss the physical limits imposed on our exposure to nanoparticles and on their detection in products and in the environment.

### The Nanomaterials Risk Debate at a Glance from a Colloidal Perspective

Despite interest having been the highest among well-educated elites, it is striking how a confluence of factors has made the debate largely driven by scaremongering newspaper headlines. This has led to a deficit with respect to what traditional physical sciences say about specific risks of nanomaterials. In this discussion note, nanomaterials will mean formulations or composites where at least one component, free or as part of a matrix, has at least one dimension in the sub-100-nm range. This is a definition that is not necessarily always scientifically and technologically important to delimit nanomaterials, but it is often used in textbooks and in legal documents.

I will not address the already immense literature dealing with cytotoxicity of nanomaterials or animal studies of toxicity of nanomaterials in a laboratory setting [1]. Even without such review, it is however fair to say that there is to date no smoking gun showing an epidemiological effect of manufactured nanoparticles on humans nor one demonstrating damage to ecosystems caused specifically by nanoparticles [1–3]. Such problems might yet arise. If they do, we might think that we acted against the danger too late if this could have been prevented by regulation. The call for specific nanomaterial regulations is based on the premise that their physical and chemical properties are starkly different from those of larger materials of the same chemical composition. Ironically, the physical properties that make nanomaterials attractive for applications are the same as the ones that raise fear of potentially higher intrinsic toxicity and environmental impact than those of traditional materials. Higher functionality is a double-edged sword that can cut in both directions if improperly used. However, it is less discussed that some of the same properties also severely limit the exposure that we should expect from nanoparticles as we encounter them in a natural (biological) environment [3].

As a scientist active in the field of nanomaterials, I see the importance and necessity of performing research on the unique properties of nanomaterials that can also lead to ecological and health problems. This has to be performed in biological and ecological systems as early as possible to detect specific risks. However, the natural laws guiding colloidal physics and chemistry dictate that any such risks will not be general for “nanomaterials,” but pertain to specific nanoparticles and applications, just as only certain chemicals and chemical uses pose a real threat to human and ecological health. Thus, nanomaterials require very extensive and detailed characterization relating to their specific harmful action to enable early risk identification and preventive measures such as “safety by design.” The required characterization, however, might not be feasible in the relevant environment, e.g., our water, our food, and our bodies. I will outline why, as a colloidal scientist, it is no surprise to me that we are “still waiting” for the smoking gun and why, despite the uniqueness of nanomaterial properties, I see it as potentially prohibitively difficult to enforce special regulation of nanomaterials in the near future.

### Are Nanoparticles a New Thing To Be Regulated?

Nanoparticles are not a modern invention. Nanoparticles are produced in natural geological and especially biological systems [2]. Natural nanoparticles include volcanic sands, viruses, and proteins. Wood fires produce huge amounts of undefined carbon nanoparticles which in their manufactured and purified forms count as some of the most advanced and useful man-made nanomaterials. Many formulations and ingredients in food, cosmetics, and pharmaceuticals, entering or in direct contact with our bodies, should be defined as nanomaterials following the standard definition employed in this article. While viruses and proteins serve as models for the design of biomedical nanoparticles such as drugs and drug delivery vehicles, they of course also highlight one of the fears concerning nanoparticles; advanced nanoparticles can travel through biological systems and have major impact on biological functions and health. However, the natural production and exposure to nanoparticles mean that evolution to a large extent has prepared us to deal with exposure to nanomaterials [2]. Thus, we might currently be increasing our exposure to man-made nanomaterials, but our contact with this class of materials is not new. The biggest concern is probably that a fraction, although in most likelihood a minor fraction, of nanomaterials will be produced from materials that have intrinsic high chemical toxicity. Examples are semiconductor quantum dots currently being produced for TV screens and optical applications. If such chemically toxic materials penetrate deeper into the body to reach sensitive tissues due to their nanoscale format, this will increase their negative impact even if no specific “nanoeffect” is causing the adverse response. However, even if we assume that nanoparticles are generally more dangerous to our health, should we automatically assume that their use leads to increased adverse exposure?

### Nanoparticles Love Each Other—You Will Not Find Them Alone for Very Long

Nanoparticles are examples of colloidal systems. Colloidal systems are characterized by the fact that one “material” is distributed in a continuous phase of another “material.” A feature of almost all colloidal systems is that the creation of the interface between the dispersed and the dispersing material costs energy. This interfacial or surface energy increases proportionally to the area between the materials. If you therefore imagine that you have only a cubic centimeter of material, creating nanoparticles of this material will cost one million times more energy than the creation of the surface of the first cube, and this energy is stored at the interface of the particles. Nature strives continuously to lower its energy. These nanoparticles will therefore, after colliding with each other and other surfaces, stick together and recombine to form larger objects. This ubiquitous process leads to the destruction over time of all nanoparticles. This might feel counterintuitive for us living in a world where we see mountains and other large structures invariably crumble to sand over time, but the balance of physical interactions is not the same for very small objects as for objects that we can see; surface interactions tend to strongly dominate other forces that could keep nanoscale objects apart.

Another inherent feature of nanoparticles is that they spontaneously move faster than their larger cousins. The energy from random thermal movement of molecules in gases and liquids transferred to an object by collisions is much larger in relation to the size (inertia) of the object for small particles. This random or Brownian motion lets nanoparticles move large distances and collide with many objects within a very short time span.

A typical suspension of nanoparticles is therefore expected to aggregate into larger objects by collision within a short time; but, how short? If the (number) concentration of particles stays the same, Brownian motion results in that the half-life time, a typical measure of the time required to lose particles from a colloidal suspension by aggregation, is the same regardless of size. Consequently, if the amount (volume or mass) of nanoparticles and large particles is the same, the half-life time decreases very rapidly with decreasing size (∝*d* ^3^) [4]. One should now ponder that, in a biological system, the synthetic nanoparticles are not the only nanoparticles present which can collide and aggregate. A natural, biological environment has a very high concentration of nanoparticles in the form of e.g. proteins in the body. The concentration of protein is so high that the half-life time of nanoparticles in such suspensions is less than milliseconds; only nanoparticles engineered to specifically avoid attractive interactions with biomolecules will not aggregate. In essence, engineering such nanoparticles is the extremely demanding problem facing every designer of biomedical nanoparticles such as drug delivery vehicles. The risk that nanoparticles made for other materials applications without special design would incidentally avoid the fate of aggregation or dissolution and thereby loss of nanoscale size in a natural environment is extremely low [3].

### When Is Nano Nano?

Why does the rapid aggregation of nanoparticles matter? Almost all specific nanorisks of materials only apply to particles that have nanoscale size. These risks relate to their fast motion and potential ability to penetrate biological tissue, their high surface area, and sometimes to the special quantum-related physical properties in terms of interacting with electromagnetic fields that nanoscale materials can have [2]. When nanoparticles aggregate, their effective size increases while their total area is reduced [3, 4]. In close proximity, the special quantum properties couple and the aggregated particles become indistinguishable from their macroscopic counterpart [5]. Thus, aggregation of just a few superparamagnetic iron oxide nanoparticles turns them into normal magnets or “rust”, and plasmonic gold nanoparticles with their enhanced optical and electric fields and size-dependent colors turn into normal yellow gold, which has been used as a biocompatible material in teeth and body jewelry for a long time. Carbon nanomaterials aggregate into bundles that are indistinguishable from common soot or the graphite in your crayon. Most importantly, as nanoparticles aggregate, deep penetration into biological organisms becomes much less probable. The increased size decreases mobility by Brownian motion; it also facilitates blocking by passive mechanisms such as cilia and narrow vasculature. Aggregation of particles with proteins (opsonins) enhances clearance by the reticuloendothelial system. Finally, also the chemical toxicity of nanoparticles will be proportional to how fast a particle is dissolved, which in turn is proportional to its free surface area and therefore is decreased by aggregation. Aggregated nanoparticles will still have a larger total surface area than their solid counterparts due to porosity. In some cases, this leads to higher catalytic activity or faster dissolution of toxic constituents than those of a solid. Biological processes during degradation of internalized materials can also theoretically deaggregate clusters of nanoparticles by active energy input. However, on balance, spontaneous and ever-present physical processes rapidly work to reduce the nanospecific risks of nanomaterials in biological systems.

It is therefore important to realize that freely dispersed colloidal nanoparticles in air or water are the ones that can pose nanospecific risks. Nanoparticles fixed in solid nanomaterials such as it is the case in computers and composite materials lead to negligible, if any, exposure, except during production and recycling of the material. At these points, special precautions can be and are taken. To set nanoparticles free from a fixed solid or even from a liquid to a gas environment takes enormous amounts of energy compared to what is available as heat in the natural environment. The natural forces driving aggregation, sedimentation, and clearance of nanoparticles, however, reduce the energy. Therefore, these processes that are destructive to nanoparticles occur spontaneously at a high rate. The net result is that an exploding use of nanomaterials is not likely to lead to a similar explosion of actual exposure of the public to potentially dangerous nanoparticles.

### Can We Prove the Presence of Nanomaterials?

If we decide that nanoparticles pose additional specific risks and merit specific regulation, we must use methods to detect and quantify their presence in situations considered to lead to potentially harmful exposure. To prove the presence of nanoparticles in a product material or in the environment, we must identify them and measure their size; to estimate nanospecific risks, we also might have to measure other properties such as surface area and shape. Any meaningful regulation of nanomaterials implicitly assumes that we can perform such measurements to enforce compliance; if not, the law cannot be enforced and possibly not even followed by companies, since they would be severely challenged to verify that they meet the regulations in their own production.

However, possibly, the idea that nanomaterials and their potential risks can easily be identified is deceptive. Nanoparticles in nanomaterials do not exist in vacuum. In most cases, they will be distributed in a solid matrix [5]; in other cases, they are dispersed in a solvent, e.g., water. Only rarely, as argued above, are we likely to encounter them in a gas phase such as air, and then most likely at the point of manufacture or at the point of destruction of the material for recycling. Thus, what methods do we have to detect and measure nanoparticles as a colloidal system within another material?

#### Tagging of Nanomaterials

Detecting the presence of nanoparticles in gases, liquids, and gels is required in e.g. environmental screening and food monitoring, which are the areas most likely to first lead to legislative concerns when the public worry about exposure to nanomaterials. So, how can we detect and measure the presence of nanoparticles in such environments? Natural biological environments are full of other small particulate matter, protein, micelles, bubbles, emulsifiers, mycobacteria, viruses, and liposomes. In nature, we further find soot, debris, and volcanic sand particles that contain nano- and submicron particles. Unfortunately, generally speaking, we lack methods to determine size and composition in a matrix simultaneously [4]. To address this, we could perceivably first separate nanoparticles from a complex environment and analyze them. Or, can we? Intriguingly, separation methods tend to depend on that we know what we are looking for to devise methods to discard other particles to be able to analyze the ones of interest. Nanoparticles often will not have a clearly distinguishing feature except for their chemical composition in a natural environment that will contain natural nanomaterials. Sometimes, as for carbon nanoparticles, even this is not true and their only distinguishing features are size and shape.

If a nanomaterial is manufactured on purpose, one suggestion that has been circulated is that they are all tagged with a reporter, like supermarket barcodes. The tag can be read out by a detector to identify and even quantify by the number of detected labels how much nanomaterial is present. Such tags could be what scientists have been using in the lab for the same purpose for ages, such as radioisotope, fluorescent, color scattering, or magnetic tags. For a moment, disregarding that also such reporters are not unique to man-made systems and any natural sample is likely to have strong optical and magnetic interactions that create a disturbing background for read-out, we should consider what introducing such labels would mean and what they can tell us. Stable tags would in themselves be nanomaterials and often be chemically toxic materials, i.e., metal (optical), doped oxide (magnetic), or semiconductor (fluorescent) nanoparticles. We would therefore require tagging a nanomaterial with another, potentially more harmful, nanomaterial. If we only look for high concentrations of nanomaterials, we could also introduce exotic (uncommon natural) elements into our products that can be detected by spectroscopic techniques, but the sensitivity of such readouts would be lower as well as more complex.

If we decide that tags should still be used, let us again consider the difference between what these detection schemes will tell us and what we would like to detect and what we aim to regulate, namely the presence of a material in its nanoparticulate form. Now, we are back to the unique feature of regulating nanomaterials compared to regulating chemicals: identifying or quantifying the presence of the chemical elements of the material does not comprise evidence of their physical state, i.e., the presence of the material in its nanoform. We will not know from quantification of the presence of the tag whether the nanoparticles are still there, have already decomposed, or if they are aggregated and do not comprise a specific nanorisk anymore.

So, instead of tagging, what methods do we have that can analyze nanoparticle size and size distribution in a liquid or gas colloidal environment? And how are they affected by sample preparation/separation or lack thereof?

#### Detection by (Light) Scattering

Nanoparticles are smaller than the wavelength of light. It is therefore not possible to resolve their size by optical microscopy or to resolve them at all if they are densely distributed in a material. Light sources and detectors are however convenient and relatively low cost and abundant. Common methods to measure nanoparticle size are therefore based on optical scattering methods, where nanoparticle size is measured based on size-specific interaction with light or from the motion of the particles in suspension, so-called dynamic light scattering. The former requires knowledge of the nanoparticle material and the environment. Its application is therefore limited to the study of samples prepared in the laboratory. The utility of both approaches is limited for polydisperse samples, for which particles of many different sizes are present. Light scattering will overemphasize large particles in a sample because its sensitivity increases as *d* ^6^, i.e., a particle that is ten times larger will give one million times more signal. These methods also only work routinely for dilute samples since each photon in the analyzed light must interact with only one single particle. The information about the size of the particle is lost if a photon goes through multiple scattering, i.e., interacts with several objects or gets absorbed and reemitted by a particle; this becomes probable at high particle concentration, although instrument developers have in recent years made clever progress to get around this problem. Notwithstanding these limitations, scattering methods have the advantage of measuring a huge number of particles simultaneously. Therefore, fitting of the data can produce reliable averages of particle sizes. While some light scattering techniques also will provide information on the shape of the particles, the most common and most easily applied technique, dynamic light scattering, will only provide information on the effective size, which is a drawback if we want to quantify shape- and area-sensitive nanoparticle properties. Scattering methods do not have to use light and can use a wave-like probe that has a shorter wavelength than visible light; this can provide advantages in terms of what information can be gained (shape, composition, etc.). However, the considerations and limitations for other high-end scattering methods such as small-angle X-ray scattering are mostly variations on those described above, although restrictions on e.g. particle concentration can be relaxed at significant cost in money and time.

It should be emphasized here that if a sample must be purified, diluted, or in any way altered to perform a measurement, we are no longer measuring the properties of the original colloidal system. Dilution and homogenization, which are applied to make a sample suitable for investigation by light scattering, strongly affect the aggregation state. It will yield a higher apparent fraction of single nanoparticles by removing aggregates and by shifting the equilibrium to a higher fraction of free nanoparticles, but it will underestimate how much of the material is in the sample if there were aggregates.

#### Detection by (Electron) Microscopy

An alternative to measure size and shape by scattering techniques is to directly inspect them using microscopy, but this requires using a probe with a wavelength sufficiently small to image nanoscale objects. Electron microscopy uses electrons instead of optical photons and therefore has a much higher resolution than light-based microscopy; it can easily resolve at least the projection of nanoscale objects. However, while light microscopy can be applied at ambient conditions, e.g., in water, high-resolution electron microscopy must be performed in vacuum. It is therefore only performed after deposition of nanomaterials on a grid and imaged in vacuum. This has the effect that, whenever we perform electron microscopy to investigate nanomaterials, we have altered the material before observing it. In the colloidal science community, such data are therefore not accepted as proof of whether particles are free or aggregated, i.e., if the particles should be considered to have their nanoscale properties or are aggregates with mainly macroscopic material properties. At best, such microscopy can give us an indication of the effective size of particles in a sample, but it can also give us additional information about the original particle shape. It can also be very challenging to identify nanoparticles of interest in a complex sample, since despite common misconceptions, only the highest end electron microscopy can resolve the chemical composition of nanoparticles near the resolution at which it images. In the dark, all cats are gray, and unfortunately nanoparticles under an electron microscope in this respect are just like cats in the dark.

Again, sample preparation demands that a dilute sample is used to prepare the grid on which the imaging is performed, since otherwise too thick a layer of material is deposited. This leads to the same inherent bias as described for the preparation of samples for light scattering. Additionally, upon drying a sample onto a grid, large nanoparticles are more likely to stick to the grid while smaller particles with weaker attraction to the grid surface are more likely to be washed away upon drying.

For application as a tool to determine regulatory compliance, electron microscopy has an additional severe drawback. Only a fraction of a sample can be inspected and, as described above, this part of the sample might not be representative for the whole population of particles. That only a small sample can be investigated is crippling to the collection of sufficient data even disregarding systematic errors derived from sample selection. This illustrates an additional reason besides costs for why scattering methods and not the more information-rich microscopy remain the core methodology to determine size and state of particles in suspension. While microscopy even with automated image analysis deals with particle statistics on the order of thousands of particles, scattering techniques will probe on the order of > 1 million particles within a fraction of the time. This is decisive because quality standards for materials characterization require that to determine a size distribution with 95% confidence for a typical synthetic nanoparticle sample requires on the order of 100,000 particles to be analyzed.

#### Nanoparticles in Solid Materials

It is likely that future high-volume nanoparticle applications will be additives to improve the performance of other materials (mechanically, optically, electrically, etc.) in solid composite materials. If it is an organic (polymer) matrix, X-ray scattering, but not light scattering, can be used to detect embedded, higher atomic number, inorganic nanoparticles. Only monodisperse nanomaterials (ones where all particles have similar size) can be size determined accurately with such techniques and it cannot be done in the bulk of the material. Similarly, electron microscopy can be applied only if submicron thin slices of a polymeric material can be cut with inorganic nanoparticles in them and the nanoparticles look sufficiently different from natural inhomogeneities in the material in terms of density and shape. Detection and quantification of inorganic nanoparticles in inorganic matrices and especially organic nanoparticles in organic matrices are thus extremely difficult with these methods; the physical interactions by which they are measured require a contrast in electron density between the nanoparticle and the surrounding matrix, which is very low for similar materials. Again, it is important to realize that these methods, which potentially can detect individual nanoparticles and even image them, do not do that with simultaneous determination of the chemical composition of the individual nanoparticles.

## Implications of the Physical Limitations of Nanoparticle Detection and Quantification

There are of course many more methods to analyze colloidal samples available, with names such as field-flow fractionation, induction-coupled plasma mass spectrometry, or atomic force microscopy, that appear in the modern literature as methods for characterization and quantification of nanoparticles. Although they use other measurement principles, they roughly face the same challenges as the separation, scattering, and microscopy methods discussed above. In particular, the problem of biasing the all-important colloidal state of the particles based on the sample preparation required to detect nanoparticles is always present. A bit pointedly, we can be said to face the situation to investigate a sample either by the components from which it is made at the start or by the components after breaking up and separating the parts of the sample; neither of those situations reflect the relevant situation. Finding the parts of ten cars in storage at the workshop and the parts of half a car in bins at the junkyard provides little information on how many functional cars are a danger to you on the street.

In the end, it is safe to say that if we take seriously that a nanoscale particle must remain non-aggregated to produce nanospecific toxic effects, we face a serious lack of methods to verify if this criterion is fulfilled in an unknown sample. In my view, this must have serious implications for how we design nanospecific regulations and communicate consumer protection measures to the public. If specific regulation of nanomaterials that requires a labeling and certification system is used as a tool to make the public feel safe, then we are setting ourselves up for failure at the current stand of metrology. We are then asking producers of food, consumer products, and everyday consumable and durable materials to do something for which they are poorly equipped and of which they have poor understanding. We are also asking them to do so at great economic cost, while—honestly—not being able to control their compliance. Even the research community with its most advanced tools will be at a loss to post facto determine whether regulations such as the recent EU nanofood directive or similar initiatives were followed during production and in the final product.

By emphasizing the properties of individual nanoparticles in public debate and legal regulations, we might also create an impression of higher danger than warranted for the uninitiated. Today, we are “still waiting” for an explosion of nanoparticle-related health problems and colloidal science tells us that dangers related to nanoparticle exposure are indeed likely to be very rare. At the same time, we are lulling the public to believe that the authorities have the perceived danger under control; however, this control is also illusionary for simple physical reasons. Even if we would massively increase our requirements on self-verification and the instrumentation available for state control of product nanosafety, we do not possess the technology that allows us to perform the characterization that a scientist would say is necessary to ensure regulatory compliance or proper risk assessment.

## Conclusion

The pipe dream of a colloidal scientist is that governments instead of promoting simplistic legislative solutions would educate the public with more help from the media to understand that although our exposure to man-made nanomaterials undoubtedly is increasing, the presence of nanomaterials is not new, neither to humans nor to the environment. This message should transmit that simple physical laws set limits for situations in which people not involved in production and recycling will be exposed to nanomaterials in their nanoparticulate form for which specific nanodangers might exist. In summary, on the positive side, although manufacturing and use of nanomaterials (defined as materials with nanoparticle components) are set to drastically increase, the physical principles governing the interactions of nanoparticles mean that the exposure of the large majority of people to nanoparticles is not set to increase proportionally. This explains to a certain extent why, both in the real world and in lab tests, the feared epidemic in nanomaterial-related health problems has not been observed. On the negative side, if society still desires to enact specific nanomaterial regulations, it will be very difficult to follow up on and enforce such laws and regulations. Techniques are missing by which we can determine the presence, quantity, and properties of nanoparticles in the environment; this is due to the complex nature of nanomaterials, i.e., their small size and strong interactions with each other and the environment. We mostly can observe nanoparticles only when we have liberated and isolated them and, thereby, in essence distorted the colloidal system and misrepresented the potential exposure. The implication is that ethical and legal debates on this subject explicitly should consider what risk assessment and control mean on a length scale that we cannot directly observe. This is didactically challenging, because the physical rules that dominate this length scale do not agree with our everyday intuition, for good as much as for bad.
